# The Capacity of *Mycobacterium tuberculosis* To Survive Iron Starvation Might Enable It To Persist in Iron-Deprived Microenvironments of Human Granulomas

**DOI:** 10.1128/mBio.01092-17

**Published:** 2017-08-15

**Authors:** Krishna Kurthkoti, Hamel Amin, Mohlopheni J. Marakalala, Saleena Ghanny, Selvakumar Subbian, Alexandra Sakatos, Jonathan Livny, Sarah M. Fortune, Michael Berney, G. Marcela Rodriguez

**Affiliations:** a Public Health Research Institute at New Jersey Medical School, Rutgers, The State University of New Jersey, Newark, New Jersey, USA; b Department of Immunology and Infectious Diseases, Harvard T. H. Chan School of Public Health, Boston, Massachusetts, USA; c Division of Immunology, The Institute of Infectious Disease and Molecular Medicine, University of Cape Town, Observatory, South Africa; d The Genomics Center at New Jersey Medical School, Rutgers, The State University of New Jersey, Newark, New Jersey, USA; e The Broad Institute, Cambridge, Massachusetts, USA; f Department of Microbiology and Immunology, Albert Einstein College of Medicine, Bronx, New York, USA; Washington University in St. Louis School of Medicine

**Keywords:** *Mycobacterium tuberculosis*, TB granuloma, iron acquisition, iron deprivation, iron sequestration, latent TB, nutritional immunity, persistence

## Abstract

This study was conducted to investigate the role of iron deprivation in the persistence of *Mycobacterium tuberculosis*. We present evidence of iron restriction in human necrotic granulomas and demonstrate that under iron starvation *M. tuberculosis* persists, refractive to antibiotics and capable of restarting replication when iron is made available. Transcriptomics and metabolomic analyses indicated that the persistence of *M. tuberculosis* under iron starvation is dependent on strict control of endogenous Fe utilization and is associated with upregulation of pathogenicity and intrinsic antibiotic resistance determinants. *M. tuberculosis* mutants compromised in their ability to survive Fe starvation were identified. The findings of this study advance the understanding of the physiological settings that may underpin the chronicity of human tuberculosis (TB) and are relevant to the design of effective antitubercular therapies.

## IMPORTANCE

One-third of the world population may harbor persistent *M. tuberculosis*, causing an asymptomatic infection that is refractory to treatment and can reactivate to become potentially lethal tuberculosis disease. However, little is known about the factors that trigger and maintain *M. tuberculosis* persistence in infected individuals. Iron is an essential nutrient for *M. tuberculosis* growth. In this study, we show, first, that in human granulomas the immune defense creates microenvironments in which *M. tuberculosis* likely experiences drastic Fe deprivation and, second, that Fe-starved *M. tuberculosis* is capable of long-term persistence without growth. Together, these observations suggest that Fe deprivation in the lung might trigger a state of persistence in *M. tuberculosis* and promote chronic TB. We also identified vulnerabilities of iron-restricted persistent *M. tuberculosis*, which can be exploited for the design of new antitubercular therapies.

## INTRODUCTION

*Mycobacterium tuberculosis* is a facultative intracellular pathogen able to survive in an infected host for decades, with little or no replication, undetectable by current diagnostic methods and causing no symptoms but capable of resuming growth and producing active tuberculosis (TB) when immune control weakens (1). This capacity of *M. tuberculosis* to persist in the host is a major obstacle to TB eradication because most antibiotics are inefficient at killing quiescent bacteria (2). The risk of reactivation of latent TB infection (LTBI) to active disease is estimated to be 5 to 10% over a lifetime (3); thus, the approximately 2 billion people predicted to have LTBI constitute an enormous reservoir of new TB cases (3). However, the host conditions that trigger quiescence, the physiology of nonreplicating bacteria, the molecular mechanisms underpinning persistence, and the pathways to reactivation of once-dormant bacilli are not well understood (4).

Like most living organisms, *M. tuberculosis* requires iron (Fe) as a redox cofactor for vital enzymes (4). However, free Fe is not available *in vivo*, and restricting pathogen access to Fe through enhanced production of Fe sequestration proteins and Fe withdrawal from circulation is a major component of the host immune defense, also known as nutritional immunity (5). To compete with the host for Fe, *M. tuberculosis* synthesizes molecules responsible for Fe binding (i.e., siderophores), heme binding, Fe transport, and Fe storage (4). There is ample evidence implicating Fe availability in TB pathogenesis. First, *M. tuberculosis* factors responsible for maintaining Fe homeostasis have been shown to be required for virulence (6–10). Second, several studies have reported that altered host Fe status is linked with TB pathogenesis (11). For instance, high macrophage Fe stores and nutritional Fe overload are associated with increased risk of developing TB and with disease exacerbation (11–13). Thus, an increase in Fe available to *M. tuberculosis* is directly linked to bacterial proliferation and acute TB. In addition, reactivation of LTBI in humans upon Fe supplementation to treat anemia, first reported more than a century ago (14, 15), suggests that Fe deficiency might sustain chronic TB infection, at least in a subset of infected individuals. Nevertheless, the relationship between the *M. tuberculosis* response to Fe deprivation and chronic TB has not been investigated.

In this study, we demonstrate that *M. tuberculosis* has the capacity to persist without growth, under conditions of strict Fe restriction such as those implemented by the human immune defense in necrotic granulomas. Employing genetic, genomic, and metabolomic approaches, we characterized the response of *M. tuberculosis* to prolonged Fe starvation and identified determinants of survival. These results expose a potential role of nutritional immunity in promoting chronic TB and provide a new view of persistent tuberculous bacilli that can provide opportunities for new host- and pathogen-directed therapeutics.

## RESULTS

### The Fe environment of human granulomas.

**FIG 1:**
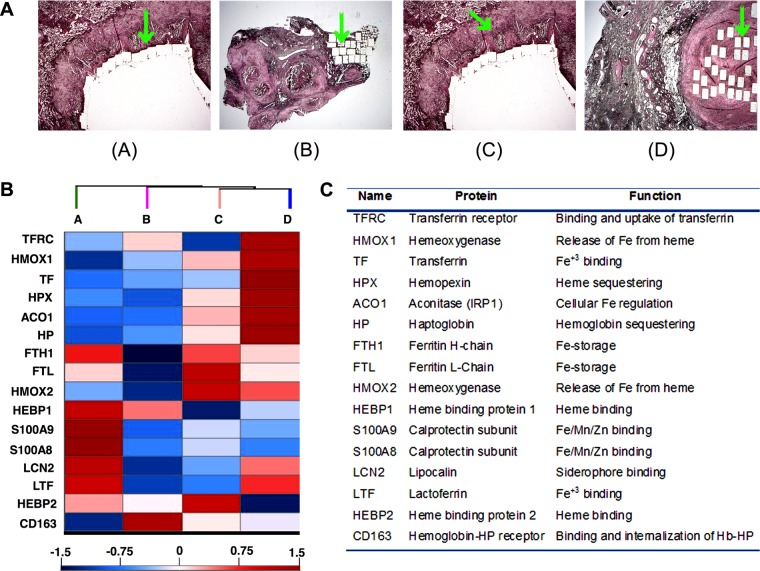
Proteomic analyses of host Fe-restricting proteins in human granulomas. (A) Histological sections of the types of granulomas sampled in the study pointing to the area dissected (green arrows): cavitary granuloma (necrotic region dissected) (A), solid granuloma (cellular region dissected) (B), cavitary granuloma (cellular region dissected) (C), and caseous granuloma (necrotic regions dissected) (D). (B) Heat map plot showing relative abundance of Fe-sequestering factors in the dissected areas of granulomas shown in panel A. (C) Proteins depicted in panel B and their function in iron metabolism.

The pathological hallmark and main stage of human TB infection is the granuloma, an inflammatory lesion that progresses from aggregates of innate immune cells to an organized structure in which the macrophage-rich center is surrounded by a lymphocytic cuff composed of T and B cells (16). As the infection progresses, some granulomas accumulate necrotic material with consequent formation of a caseum at the center, which may undergo liquefaction, sometimes resulting in the formation of a cavity (Fig. 1) (17). Granulomas evolve independently of each other, and different compartments within single granulomas exhibit molecular heterogeneity (18, 19). Therefore, to examine the Fe environment of human granulomas, we analyzed a published data set (19) for the relative abundance of host Fe-sequestering proteins, as indicators of Fe bioavailability in histologically distinct compartments of granulomas, which were separated by laser-guided microdissection and assayed by quantitative mass spectrometry (MS) (19) (Fig. 1). The relative abundance of proteins was based on quantification of a protein in a given dissected histological region compared to all other samples subjected to proteomics. These analyses showed that CD163, the cell surface receptor that mediates internalization of hemoglobin (Hb) (20); heme binding proteins (HEBP1 and -2); and soluble transferrin receptor (TFRC) were abundant in solid cellular granulomas (Fig. 1). In addition, the cellular regions of cavitary granulomas show a high abundance of ferritin (FTH/FL) and heme oxygenase (HMOX). Collectively, these are indicators of the cellular engagement in Fe uptake, Hb and heme-Fe recycling, and intracellular Fe storage, suggesting that *M. tuberculosis* may find a permissive Fe environment within these cells. In contrast, the necrotic center of a caseous granuloma showed accumulation of transferrin (TF), haptoglobin (HP), and hemopexin (HPX) (Fig. 1), which are potent extracellular sequesterers of Fe^3+^, Hb, and heme, respectively. Also abundant in necrotic areas were Fe-restricting antimicrobial proteins released by neutrophils upon degranulation, like Fe binding lactoferrin (LTF) (21) and lipocalin (LCN2), which sequesters siderophores and is involved in inhibition of mycobacterial growth *in vivo* (22, 23), and the metal chelator calprotectin (S100A9/S100A8) (24–26) (Fig. 1). Data analysis of an independent study of host gene expression in human granulomas compared to uninvolved tissue (27) showed a direct correlation between gene induction and abundance of Fe-sequestering proteins detected in the proteome of necrotic and cavitary granulomas, thus supporting an enhanced host Fe-restricting response in advanced TB granulomas (see Fig. S1 in the supplemental material). The concentration of basically all known Fe-restricting host proteins in the necrotic center of advanced granulomas suggests that bacteria might be subjected to intense Fe deprivation in this microenvironment. The high induction of Fe acquisition genes previously observed in *M. tuberculosis* isolated from infected human lungs is consistent with this interpretation (28).

10.1128/mBio.01092-17.1 FIG S1  Host Fe metabolism gene expression in TB cavitary granulomas. Heat map representing abundance of transcripts of genes encoding the indicated Fe metabolism proteins in the cellular region adjacent to necrotic-cavitary (Cav) granulomas compared to uninvolved regions isolated from the lungs of human TB patients. TFRC, transferrin; HMOX1 and -2, heme oxygenase; TF, transferrin; HPX, hemopexin; ACO1, aconitase; FTH1, ferritin heavy chain; FTHL, ferritin light chain; HEPB1 and -2, heme binding protein; S100A9/8, calprotectin; LCN2, lipocalin; LTF, lactoferrin; CD163, haptoglobin-hemoglobin receptor. Acquisition of lung tissue and processing of the tissue for microarray analysis are described in Materials and Methods. Download FIG S1, PDF file, 0.1 MB. Copyright © 2017 Kurthkoti et al. 2017 Kurthkoti et al. This content is distributed under the terms of the Creative Commons Attribution 4.0 International license.

### *M. tuberculosis* persists under Fe starvation.

**FIG 2:**
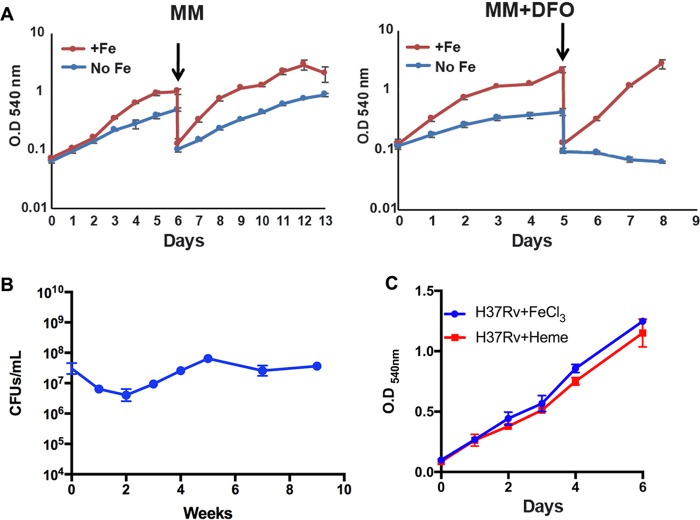
Iron starvation and persistence of *M. tuberculosis*. (A) *M. tuberculosis* was sequentially cultured in MM with FeCl_3_ (+Fe) or in MM without Fe (No Fe) and MM+DFO. Growth was monitored based on the increase in optical density at 540 nm (OD_540_). Black arrows indicate the time when the cultures were diluted. (B) Shown is the number of CFU per milliliter recovered after plating serial dilutions of MM+DFO cultures at indicated time points onto 7H10. (C) Fe-starving *M. tuberculosis* growth after culture supplementation with FeCl_3_ or heme. Error bars represent means ± standard deviations from three biological replicates.

Considering the likely Fe deprivation of *M. tuberculosis* in necrotic granulomas, we examined how *M. tuberculosis* responds to conditions of strict Fe starvation. We established an *in vitro* model where *M. tuberculosis* was recovered from a frozen stock in standard 7H10 agar and transferred into Fe-depleted defined minimal medium (MM) and MM supplemented with deferoxamine (MM+DFO), a heterologous siderophore that chelates Fe^3+^ in the medium but is not taken up by *M. tuberculosis* (29). While the *M. tuberculosis* growth rate in MM was slightly reduced relative to cultures supplemented with FeCl_3_, the cultures continued growing under these Fe-deficient conditions (Fig. 2A), possibly using traces of Fe in the medium and Fe stored within the cell (Fig. 2A). In contrast, the first pass of *M. tuberculosis* into MM+DFO resulted in a decreased growth rate, and the second pass into the same medium fully arrested *M. tuberculosis* growth (Fig. 2A). Although these Fe-starved mycobacteria ceased to grow, CFU enumeration by plating onto standard Fe-rich 7H10 medium showed that the cultures remained viable for over 9 weeks, the last time point examined (Fig. 2B). Furthermore, when the cells were provided with FeCl_3_ or heme, they resumed normal replication (Fig. 2C). This remarkable ability to persist in the absence of exogenous Fe was not unique to *M. tuberculosis* H37Rv: other strains (Table 1), including *M. tuberculosis* Erdman and *M. tuberculosis* CDC1551, a recent clinical isolate (30), behaved similarly (Fig. S2).

10.1128/mBio.01092-17.2 FIG S2  *M. tuberculosis* CDC1515 (A) and *M. tuberculosis* Erdman (B) persistence under Fe starvation. Shown is the number of CFU per milliliter recovered from MM+DFO cultures. Data are expressed as the means ± standard deviations from three biological replicates. Download FIG S2, PDF file, 0.1 MB. Copyright © 2017 Kurthkoti et al. 2017 Kurthkoti et al. This content is distributed under the terms of the Creative Commons Attribution 4.0 International license.

**TABLE 1:** *M. tuberculosis* strains used in this study

*M. tuberculosis* strain	Description	Source or reference
H37Rv	*M. tuberculosis*	ATCC
Erdman	*M. tuberculosis*	ATCC
CDC1551	*M. tuberculosis*	30
H37Rv *dosR* mutant	Δ*dosR*::Kan	34
H37Rv *mprA* mutant	*mprA*::Kan	I. Smith, unpublished data
H37Rv *sigE* mutant	*sigE*::Kan	72
H37Rv *sigB* mutant	*sigB*::Kan	85
H37Rv *bfrA* mutant	Δ*bfrA*::Hyg	9
H37Rv *bfrB* mutant	Δ*bfrB*::Hyg	9
H37Rv *clpB* mutant	*ΔclpB*::Hyg	56

**FIG 3:**
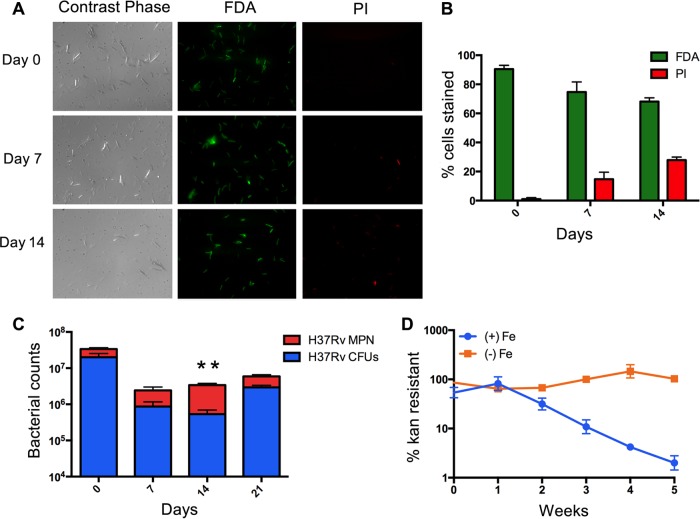
Phenotypic characterization of Fe-starving *M. tuberculosis*. (A) FDA and PI viability staining of growth-arrested MM+DFO *M. tuberculosis* cultures. (B) Quantification of the number of cells stained with FDA and PI. (C) Number of viable bacteria determined by MPN and CFU methods in MM+DFO cultures at indicated time points. (D) Percentage of Fe-starved growth-arrested *M. tuberculosis* that retained kanamycin resistance conferred by the replication clock plasmid PBP10. Error bars represent means ± standard deviations from three biological replicates. *, *P* value < 0.05; **, *P* value < 0.01.

During the first 2 weeks after growth cessation in MM+DFO, the number of CFU decreased 10- to 50-fold (Fig. 2B). However, at the same time the majority (~70 to 80%) of the cells stained positive for fluorescein diacetate (FDA) and negative for propidium iodine (PI) (Fig. 3A and B). FDA is a cell-permeant esterase substrate that measures both enzymatic activity, which is required to activate its fluorescence, and cell membrane integrity, which is required for intracellular retention of its fluorescent product. In contrast, the DNA-intercalating agent PI cannot pass through an integral cell membrane (Fig. S3). Thus, the results indicated that most Fe-starving cells were metabolically active and had an intact cell envelope. To address this discrepancy in viability measurements produced by different methods, we employed the most probable number (MPN) dilution culture method (31) and enumerated bacteria that grow in 7H9 broth. In agreement with the viability staining, the MPN method showed a larger number of viable cells than the CFU method (Fig. 3C), indicating that the MM+DFO culture contained a population of bacteria that remained viable but failed to produce colonies on 7H10 agar. Such differentially culturable bacterial cells (DCCs) have been detected during stress as well as in sputum produced by TB patients (32). However, to the best of our knowledge, this is the first time that *M. tuberculosis* DCCs have been associated with Fe deficiency.

10.1128/mBio.01092-17.3 FIG S3  Validation of FDA/PI viability staining. Mycobacterial cells were subjected to 80°C for 0, 5, or 10 min (T0, T5, and T10, respectively) and stained with FDA or PI. While unstressed viable cells stain with FDA, they do not stain with PI. Heat-killed cells are stained by PI but not by FDA. Download FIG S3, PDF file, 0.7 MB. Copyright © 2017 Kurthkoti et al. 2017 Kurthkoti et al. This content is distributed under the terms of the Creative Commons Attribution 4.0 International license.

After the 2nd week of culture in MM+DFO, the size of the DCC population decreased while the CFU numbers increased and stabilized (Fig. 2B and 3C). We termed this population “iron-starved growth-arrested *M. tuberculosis*” (ISGAM). We found that ISGAM showed enhanced tolerance to several antibiotics, including isoniazid (INH), a first-line TB drug (Table 2). However, they remained as equally sensitive to rifampin as Fe-sufficient bacteria, indicating that DNA transcription is required for acclimation to Fe starvation.

**TABLE 2:** Differential antibiotic sensitivity in Fe-starving growth-arrested *M. tuberculosis* and Fe-sufficient bacteria

Antibiotic	MBC_90_ (μg/ml) on culture^a^:	
	MM+DFO	MM plus Fe
Ciprofloxacin	>16	1
Cycloserine	>200	12.50
Ethionamide	>40	1.20
Isoniazid	10	0.30
Kanamycin	>25	6.20
Rifampin	0.06	0.06

^a^ The MBC_90_ was determined for biological triplicates of MM+DFO and MM-plus-Fe *M. tuberculosis* cultures.

To assess whether ISGAM was equivalent to a nonreplicative state, we used a “replication clock”—an unstable plasmid that is lost at a constant rate per cell division (33). We found that ISGAM retained the plasmid (as determined by resistance to kanamycin) for up to 5 weeks, indicating very little or no replication (Fig. 3D). Collectively, these results demonstrate that the *M. tuberculosis* response to Fe deprivation—a condition that it might encounter in necrotic granulomas –is dynamic and includes phases of cell death, differential culturability, and nonreplicative persistence.

### **Transcriptional and metabolic responses of Fe-starving** *M. tuberculosis***.**

**FIG 4:**
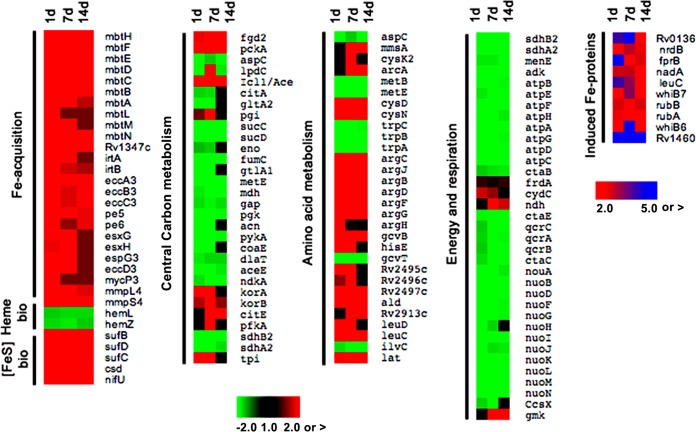
Time course transcriptional profile of Fe-starved *M. tuberculosis*. Triplicate *M. tuberculosis* cultures from three independent experiments were harvested at days 1, 7, and 14, and RNA was extracted. *P* values for heat maps can be found in the GEO database (accession number GSE84554).

ISGAM’s transcriptional and metabolic profiles were analyzed to investigate the mechanisms of persistence under Fe starvation. Differential gene expression detected in microarrays (Fig. 4) was generally confirmed by transcriptome sequencing (RNA-seq) (Fig. S4 and Data Set S1D). Although growth-arrested cells repressed macromolecular synthesis and growth genes, a few hundred gene transcripts encompassing several functional categories (Table S1) were more abundant in ISGAM than in Fe-replete cells (≥2.0-fold change, with a false discovery rate [FDR] of ≤0.01). However, over 20% of induced genes encoded proteins of unknown function, highlighting a yet-unknown component of the Fe starvation response.

10.1128/mBio.01092-17.4 FIG S4  Heat map based on RNA-seq of selected differentially expressed genes detected in microarrays. Triplicate cultures were harvested at days 1 and 7, and RNA was extracted and processed for RNA-seq as described in Materials and Methods to validate the microarrays. *P* values adjusted for multiple testing using the Benjamini-Hochberg procedure (P_adj_) are shown in Data Set S1. Download FIG S4, PDF file, 0.1 MB. Copyright © 2017 Kurthkoti et al. 2017 Kurthkoti et al. This content is distributed under the terms of the Creative Commons Attribution 4.0 International license.

10.1128/mBio.01092-17.8 TABLE S1 Functional categories of genes upregulated during the Fe starvation response (ISR). Download TABLE S1, PDF file, 0.2 MB. Copyright © 2017 Kurthkoti et al. 2017 Kurthkoti et al. This content is distributed under the terms of the Creative Commons Attribution 4.0 International license.

10.1128/mBio.01092-17.9 DATA SET S1  (A) List of genes induced in Fe-starved *M. tuberculosis* that were also found induced in *M. tuberculosis* subjected to hypoxic conditions in the study by Park et al. (34). (B) List of genes induced in Fe-starved *M. tuberculosis* that were also found induced in *M. tuberculosis* subjected to nutrient starvation in the study by Betts et al. (35). (C) List of genes induced in *M. tuberculosis* under Fe starvation but not in *M. tuberculosis* subjected to hypoxia or nutrient starvation. (D) List of genes differentially expressed in ISGAM microarrays validated by RNA-seq. Download DATA SET S1, XLSX file, 0.05 MB. Copyright © 2017 Kurthkoti et al. 2017 Kurthkoti et al. This content is distributed under the terms of the Creative Commons Attribution 4.0 International license.

A comparison of the ISGAM transcriptional profile with those reported for nonreplicating *M. tuberculosis* under oxygen depletion (34) and nutrient-starved *M. tuberculosis* (35) and with the common expression profile of those conditions and stationary-phase *M. tuberculosis* (36) identified 124 genes commonly induced in Fe starvation and at least one other growth-limiting condition (Data Set S1A and B), while ~240 genes were induced only in Fe starvation (Data Set S1C). Thus, with the caveat that parallel experimental comparisons are the best way to define a condition-specific gene signature, the *M. tuberculosis* response to Fe starvation appears to have a unique gene expression component that is also reflected in the metabolic activity of ISGAM as described below.

### (i) Economics of Fe in ISGAM.

Fe-starving bacteria maintained a high abundance of Fe-acquisition gene transcripts for at least 14 days, the last time point examined, indicating that they continued trying to obtain Fe (Fig. 4). They also upregulated essential Fe-S cluster [Fe-S] assembly genes (*suf*) (37) while reducing synthesis of heme and most, but not all, Fe proteins (Fig. 4). These changes are consistent with an active Fe-sparing response—a coping strategy in which cells prioritize the use of scarce Fe by repressing the synthesis of dispensable Fe proteins (38). Thus, the *M. tuberculosis* Fe-sparing response seems to prioritize [Fe-S] biogenesis over heme synthesis and synthesis of Fe proteins involved in electron transfer (Cyp138, FprB, RubA, and RubB), NAD synthesis (NadA), and the [Fe-S]-dependent regulators WhiB6 and WhiB7 (39–42) (Fig. 4).

### (ii) Metabolic challenges associated with Fe restriction.

**FIG 5:**
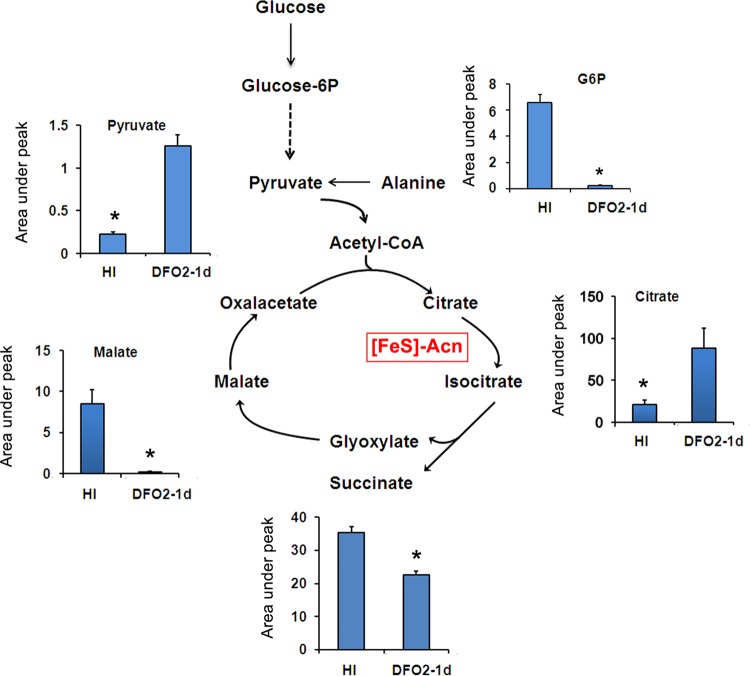
*M. tuberculosis* metabolic reprogramming during Fe starvation. Diagram showing abundance of intermediate metabolites of glycolysis, TCA, and glyoxylate cycle in Fe-replete cultures (HI) and MM+DFO cultures at 1 day into Fe starvation (DFO2-1d). The values represent the areas under the peak normalized to cell number in each sample. Error bars represent the SDs from biologically independent triplicates. *, *P* value < 0.05. CoA, coenzyme A.

Both the transcriptional and metabolomic profiles indicated that amino acid and central carbon metabolism were significantly affected by Fe starvation (Fig. 4; Table 3). Genes encoding enzymes of the tricarboxylic acid (TCA) cycle and oxidative phosphorylation were mostly repressed, which is not unexpected given the number of [Fe-S]/heme proteins involved in these pathways (Fig. 4). Concomitantly, expression of *atp* genes (encoding F_1_F_o_ ATP synthase) was decreased (Fig. 4), indicating reduced respiration-associated ATP synthesis. Furthermore, we found that ISGAM repressed expression of aconitase, the [Fe-S] enzyme that catalyzes the isomerization of citrate to isocitrate, the substrate for the TCA cycle and the glyoxylate shunt (GS) (Fig. 4 and 5). Consistent with a metabolic roadblock at this step, ISGAM accumulated high levels of citrate (Fig. 5). We also observed a decrease in cellular levels of glucose-6-phosphate and repression of glycolytic genes (Fig. 4 and 5), indicating a downregulation of glycolysis. Nevertheless, pyruvate levels increased in ISGAM (Fig. 5), suggesting a different origin for pyruvate. One possible source is alanine, since alanine dehydrogenase (Ald), the enzyme that produces pyruvate from Ala with concomitant reduction of NAD to NADH (43), was highly upregulated in ISGAM (Fig. 4).

**TABLE 3:** Metabolite abundance of Fe-starving and Fe-sufficient *M. tuberculosis*

Process and metabolite	Fold change, −Fe/+Fe^a^
Glycolysis	
Glucose-6-phosphate	0.02
Pyruvate	5.50
TCA cycle	
Citrate	4.10
α-Ketoglutarate	1.60
Succinate	0.60
Fumarate	2.90
Malate	0.02
Amino acid metabolism	
Threonine	0.30
Lysine	2.10
Aminoadipate	9.10
*N*-Acetyl-lysine	2.40
Methionine	0.04
*S*-Adenosylmethionine (SAM)	0.20
Glutamate	0.13
*N*-Acetyl-ornithine	21
Ornithine	1.80
Citrulline	0.09
Arginine	2.10
Valine	1.20
Histidine	0.50

^a^ Values correspond to the average metabolite ratio between 1-day MM+DFO (−Fe) and 1-day Fe-replete cultures (+Fe). The *P* value of the fold change for all metabolites in the table is <0.05.

Consistent with downregulation of the methionine biosynthetic genes *metC*, *metB*, *metE*, and *metA* (Fig. 4), the levels of methionine and the methionine derivative *S*-adenosylmethionine (SAM) decreased (Table 3), suggesting impaired SAM-dependent catalysis in Fe-starving *M. tuberculosis*.

While the levels of most amino acids decreased in ISGAM, lysine, which serves as a siderophore building block (44), and arginine increased (Table 3). The increase in arginine paralleled the upregulation of the entire *arg* gene cluster (Fig. 4), a decrease in the precursor amino acid glutamate, and an increase in other metabolites of this pathway, including *N*-acetyl-ornithine, ornithine, and fumarate (Table 3) (45).

Genes encoding enzymes involved in mycolic acid and cell wall dimycocerosate ester (DIMs) biosynthesis were downregulated, while genes necessary for cholesterol catabolism (46) and persistence in the host (47) were upregulated in ISGAM (Fig. S5A). ISGAM cells also stained positive with Nile red, suggesting accumulation of neutral lipids during Fe starvation (Fig. S5B).

10.1128/mBio.01092-17.5 FIG S5  (A) Heat map transcriptional profile of lipid metabolism genes in Fe-starved *M. tuberculosis*. Triplicate cultures from three independent experiments were harvested at days 1, 7, and 14, and RNA was extracted. Genes involved in cholesterol catabolism are marked with asterisks. *P* values for heat maps can be found in the GEO database (accession number GSE84554). (B) Microscopic images representative of auramine and Nile red staining of Fe-starving *M. tuberculosis* at indicated time points. MM, first passage in MM; MM+DFO1, first passage in MM+DFO; MM+DFO2, second passage in MM+DFO. (C) Percentage of Nile red-stained cells in at least 500 cells counted. Data are expressed as the means ± standard deviations from three biological replicates. Download FIG S5, PDF file, 0.4 MB. Copyright © 2017 Kurthkoti et al. 2017 Kurthkoti et al. This content is distributed under the terms of the Creative Commons Attribution 4.0 International license.

### Genetic determinants of ISGAM viability and recovery.

We next examined survival and recovery of *M. tuberculosis* gene knockout mutants to identify genes and pathways important for survival or recovery from Fe starvation.

### (i) Regulators of gene expression.

**FIG 6:**
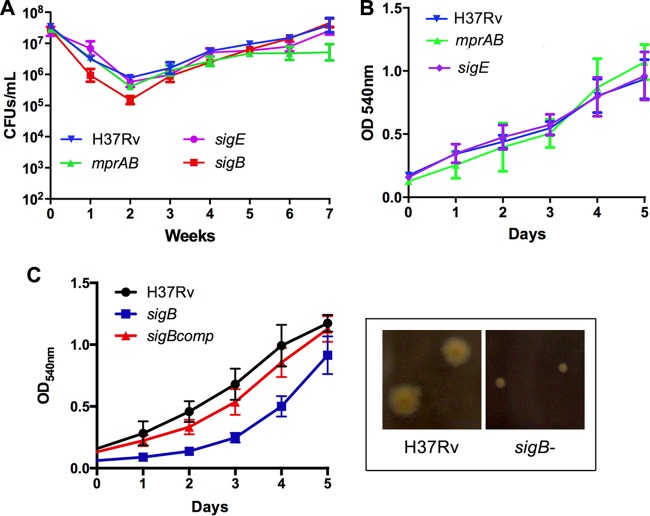
Persistence of *mprA*, *sigE*, and *sigB M. tuberculosis* mutants under Fe starvation. (A) Shown is the number of CFU per milliliter recovered after plating serial dilutions of MM+DFO cultures of *M. tuberculosis* mutant strains onto 7H10 agar. (B) Growth of Fe-starving *mprA* and *sigE* mutant *M. tuberculosis* strains supplied with Fe. (C) Growth of Fe-starving *sigB M. tuberculosis* mutant upon Fe addition The micrograph shows same-age colonies produced by *M. tuberculosis* wild type and *sigB* mutant on 7H10 agar. Data are expressed as the means ± standard deviations from three biological replicates.

The transcriptomic analyses showed the cell surface stress response network composed of the two-component system MprAB and the sigma factors SigE and SigB (48) being induced during Fe starvation, suggesting surface stress secondary to Fe deficiency in *M. tuberculosis*. Inactivation of *mprA* or *sigE* did not affect survival or recovery from Fe starvation, but the Fe-starved *sigB* mutant produced significantly smaller colonies on 7H10 and its recovery in Fe-supplemented MM was delayed (Fig. 6), indicating *mprAB*- and *sigE*-independent *sigB* expression during Fe deficiency and a role for the *sigB* regulon during recovery from Fe starvation.

A distinctive feature in the transcriptional response to Fe starvation was low or no induction of the majority of genes in the dormancy regulon, approximately 50 genes induced under oxygen limitation or exposure to nitric oxide and activated by the dormancy survival regulator (DosR) (34, 49, 50). Accordingly, a *dosR* mutant (34) survived and recovered from Fe starvation normally (Fig. S6), confirming the dispensability of the DosR regulon for persistence in the absence of Fe.

10.1128/mBio.01092-17.6 FIG S6  Survival and recovery of a *dosR* mutant subjected to Fe starvation. (A) CFU recovered from MM+DFO cultures of wild type and Δ*dosR* mutant at indicated time points into Fe starvation. (B) Growth measured as the increase in optical density at 540 nm of wild-type and Δ*dosR* strains when provided with FeCl_3_. Error bars represent means ± standard deviations from three biological replicates. Download FIG S6, PDF file, 0.3 MB. Copyright © 2017 Kurthkoti et al. 2017 Kurthkoti et al. This content is distributed under the terms of the Creative Commons Attribution 4.0 International license.

### (ii) Fe storage proteins.

**FIG 7:**
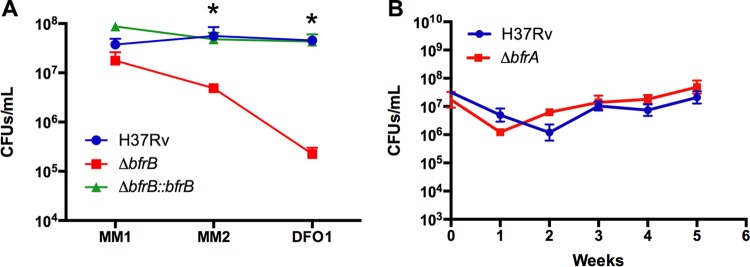
Survival of Fe storage mutants under Fe starvation. (A) CFU of wild-type and *ΔbfrB* and *ΔbfrB*-complemented strains recovered in the initial phases of Fe depletion in MM and the first passage in MM+DFO. Wild-type and *bfrB*-complemented mutant strains grew during each passage; therefore, the CFU number in the graph was obtained by plating dilutions of cultures adjusted to an OD_540_ of 0.1. (B) CFU of wild-type and Δ*bfrA* mutant in MM+DFO. Error bars represent means ± standard deviations from three biological replicates. *, *P* value < 0.05.

It has been recognized that in early stages of infection within naive macrophages, *M. tuberculosis* manipulates the phagosome to gain access to incoming Fe and that it obtains sufficient Fe via siderophores for replication and storage (9, 51–54). In our model, bacteria are recovered from frozen stocks in Fe-containing 7H10 agar, and therefore, they too can store Fe. *M. tuberculosis* encodes two iron storage proteins, a heme-containing bacterioferritin (BfrA) and a ferritin-like protein (BfrB). BfrB seems to be the housekeeping Fe storage protein in *M. tuberculosis*, while BfrA is needed under some stress conditions (9). BfrB is also needed for *M. tuberculosis* survival during the chronic phase of infection in mice, and a *bfrB* mutant shows hypersensitivity to oxidative stress and antibiotics (9). We investigated the requirement of stored Fe for acclimation to Fe starvation using isogenic *bfrA* and *bfrB* mutant strains of *M. tuberculosis*. A ferritin mutant (Δ*bfrB*) showed an ~3-log loss of viability during the initial phases of Fe depletion in MM, while a bacterioferritin mutant (Δ*bfrA*) was not affected (Fig. 7), indicating that *M. tuberculosis* depends on Fe stored in ferritin to survive. This result suggests that the size of the Fe storage pool may be a determinant of whether the bacteria can successfully adjust to Fe starvation.

### (iii) Regulators of proteostasis.

**FIG 8:**
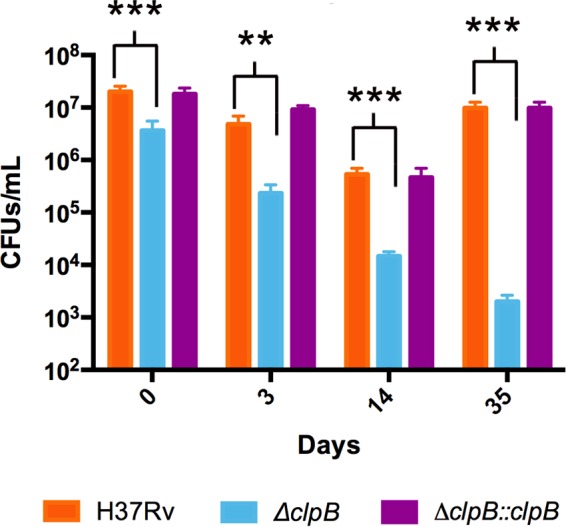
Survival of a *clpB* mutant subjected to Fe starvation. Shown are the CFU of wild-type, *ΔclpB* mutant, and *ΔclpB*-complemented *M. tuberculosis* MM+DFO cultures at indicated times. Error bars represent means ± standard deviations from three biological replicates. *, *P* value < 0.05; **, *P* value < 0.01; ***, *P* value < 0.001.

Our transcriptomics and metabolomic analyses suggested that efficient recycling and reuse of Fe from nonessential proteins and essential protein recycling might be critical for enduring Fe starvation. To test this prediction, we interrogated the transcriptomics data for upregulated genes encoding proteins that might facilitate these processes. We found that *clpB*, which encodes a chaperone that helps disaggregate misfolded proteins and sequesters those that are irreversibly damaged (55, 56), was highly induced in ISGAM at all time points tested (2.1-, 4.5-, and 5.6-fold at 1, 7, and 14 days, respectively). Therefore, we examined the effect of *clpB* inactivation (56) on *M. tuberculosis* persistence. CFU recovered from the *clpB* mutant decreased drastically during Fe starvation (Fig. 8), suggesting an important role for ClpB-mediated proteostasis in enduring Fe deprivation.

## DISCUSSION

This study showed that the necrotic center of human granulomas has a high concentration of Fe-sequestering proteins and Fe-restricting factors, likely establishing an Fe-deprived environment for the infecting *M. tuberculosis*. We also showed that *M. tuberculosis* has a remarkable ability to persist for a long time under conditions of Fe deprivation with little or no replication, refractive to antibiotics and capable of resuming normal growth when Fe is restored. Our findings raise the possibility that an effective nutritional immunity might also promote establishment of chronic TB. This expands the current view of the host responses to infection that might trigger quiescence of *M. tuberculosis* and provides new opportunities to identify metabolic vulnerabilities of persistent bacteria that can be targeted for new therapeutics.

Our results revealed that Fe starvation triggers the appearance of bacilli that, although viable, were unable to form colonies on agar plates. *M. tuberculosis* isolates exhibiting a similar differentially culturable cell (DCC) phenotype have been detected in sputum samples from TB patients prechemotherapy (57), indicating that they occur *in vivo* and are therefore extremely important in culture-based diagnostics and evaluation of treatment efficacy. Nonetheless, they are poorly characterized due in part to the difficulties involved in detecting them. Since DCCs emerge in a highly reproducible manner in Fe-starving cultures, the *in vitro* model established here could be valuable to further understand the physiology of this bacterial population. In addition, our results suggest a high probability that some transmitted bacilli are Fe deficient. This might influence initial host pathogen interactions as infecting bacilli must regain access to Fe to establish a new productive infection. Indeed, we found that Fe deficiency induces expression of genes needed for subversion of macrophage antimicrobial defense, such as the type VII secretion system Esx-3, and the protein kinase PknG and other virulence-associated factors, including sulfolipid biosynthesis, cholesterol utilization genes, isocitrate lyase, phosphoenolpyruvate carboxykinase, and WhiB6 (47, 58–61). Collectively, these observations support the idea that *M. tuberculosis* coordinates the response to Fe limitation with expression of factors that enable survival in the host.

Fe-starving growth-arrested *M. tuberculosis* shows reduced sensitivity to several antibiotics, including the first-line TB drug isoniazid (INH), also used in preventive therapy against LTBI reactivation. One explanation for this observation is that nongrowing cells contain a reduced abundance of the drug target. However, Fe deficiency may also specifically contribute to antibiotic tolerance. For instance, INH is a prodrug activated by catalase (KatG), a heme enzyme (62, 63). Since *katG* is downregulated ~2.0-fold under Fe starvation, reduced drug activation may contribute to ISGAM’s INH tolerance. In addition, inhibition of the electron transport chain due to Fe deficiency likely mediates enhanced tolerance to aminoglycoside antibiotics, which require an active electron transport chain to enter the cell. Furthermore, the genes encoding recognized intrinsic antibiotic resistance enhancers, such as WhiB6 (40), WhiB7 (42), and the fluoroquinolone efflux pump encoded by Rv2688c (64), are also induced in response to Fe deficiency, supporting an active role of the Fe-deficiency response in antibiotic tolerance. Given these results, we believe that Fe-limited *M. tuberculosis* constitutes a valuable screening platform for new antitubercular antibiotics.

In trying to understand the essential metabolic reprogramming of Fe-starving *M. tuberculosis*, we sought out parallels with *Lactobacillus* sp. and *Borrelia burgdorferi*, which are exceptional in that they exist and proliferate without Fe (65, 66). These bacteria have no [Fe-S] assembly genes, and the *B. burgdorferi* genome does not encode enzymes of the TCA cycle or oxidative phosphorylation—pathways that depend largely on [Fe-S]/heme proteins. Both of these bacteria rely on substrate phosphorylation for production of ATP (66) and depend on pyruvate conversion to lactate via lactate dehydrogenase to regenerate NAD^+^ required for continued glycolysis. Metabolic detection of lactate in ISGAM suggests that *M. tuberculosis* performs homolactic fermentation during Fe starvation; however, further analyses are needed to decipher the metabolic sources of energy in ISGAM. *Lactobacillus* and *Borrelia* also use Mn^2+^ instead of Fe^2+^ as a cofactor of several essential enzymes. Mycobacteria also show indications of employing this strategy. For instance, the superoxide dismutase SodC, which is essential to resist exogenous oxidative stress, is a Cu,Zn enzyme (67). Thus, it is possible that over its long coexistence with humans experiencing Fe limitation, *M. tuberculosis* has evolved cambialistic enzymes and/or learned to use alternative available cofactors.

Interestingly, we found that ISGAM did not accumulate succinate, which is accumulated in *M. tuberculosis* under hypoxic conditions (68). Secretion of succinate, derived from isocitrate cleavage, is thought to help *M. tuberculosis* maintain a proton motive force under respiratory stress caused by oxygen depletion (68, 69). Instead, we found that citrate and fumarate increase in Fe-starving *M. tuberculosis* (Fig. 5), suggesting that ISGAM and oxygen-depleted nonreplicative *M. tuberculosis* use distinct mechanisms to maintain an energized membrane. The specific nature of these mechanisms is under investigation.

Our findings regarding the marked increase in arginine biosynthesis during Fe starvation are very intriguing, particularly in the context of recent discoveries of the role of arginine as a metabolic regulator of survival of activated T cells (70) and as a metabolic cue in intraspecies communication in bacteria. l-Ornithine, an arginine synthesis intermediate exported by *Enterococcus faecalis*, a pathogen involved in polymicrobial pathogenesis, stimulates *Escherichia coli* to synthesize enterobactin, a siderophore needed for biofilm formation under iron-limiting conditions (71).

This study identified two cellular processes important for persistence under Fe starvation: Fe storage in ferritin (BfrB) and ClpB-mediated proteostasis. The Δ*bfrB* survival defect suggests that shutting down growth before Fe reserves in ferritin are exhausted is necessary to maintain viability during Fe deprivation. Thus, it is possible that a reduced Fe-ferritin pool may signal poor nutritional conditions leading to activation of cell growth control mechanisms in *M. tuberculosis*. BfrB and ClpB were previously shown to be important also for *M. tuberculosis* replication *in vivo* (9, 56). Together with our study, these results indicate that these proteins constitute good targets for developing antimicrobials effective against both replicating and persistent *M. tuberculosis*.

The World Health Organization estimates indicate that 1.6 billion people, over 25% of the world’s population, suffer anemia, mainly due to Fe deficiency (86). These numbers, the adverse effects of Fe repletion on the course of TB, and the results of this study underscore the need for careful monitoring of Fe supplementation programs in areas where TB is endemic. Collectively, they also lend support to the intriguing theory that Fe deficiency may be part of an evolutionary compromise permitting optimum coexistence of host and pathogen (15).

## MATERIALS AND METHODS

### Bacterial strains, media, and growth conditions.

Bacterial strains and plasmids used in this study are listed in Table 1. *M. tuberculosis* strains were recovered from frozen stocks on Middlebrook 7H10 agar (Difco) supplemented with 0.5% (vol/vol) glycerol, 0.05% Tween 80, and 10% (vol/vol) albumin-dextrose-NaCl complex (ADN). Fe-depleted minimal medium (MM) was used for iron starvation experiments. MM contains 0.5% (wt/vol) asparagine, 0.5% (wt/vol) KH_2_PO_4_, 2% glycerol, 0.05% Tween 80, and 10% ADN, and its pH was adjusted to 6.8 with NaOH. To remove metal ions, MM was treated with 5% Chelex-100 (Bio-Rad) for 24 h at 4°C with gentle agitation. The Chelex-100 resin was removed by filtration through a 0.22-μm filter (Millipore), and the medium was supplemented with 0.5 mg of sterile ZnCl_2_ liter^−1^, 0.1 mg of MnSO_4_ liter^−1^, and 40 mg of MgSO_4_ liter^−1^. The amount of residual Fe in this medium, determined by atomic absorption spectroscopy, is ~1 µM. The *M. tuberculosis mprA* mutant used in this study was a kind gift from Issar Smith (PHRI, Newark, NJ), and it was obtained by insertion of a Kan^r^ cassette (*aph*) in the *mprA* gene via homologous recombination using the suicide plasmid PSM270 (72). Disruption of the gene was confirmed by PCR analysis (see Fig. S7 in the supplemental material).

10.1128/mBio.01092-17.7 FIG S7  Confirmation of *mprA*::Kan mutant strain. (A) Schematic of the insertion of kanamycin cassette within *mprA*. (B) PCR amplification in the wild-type H37Rv and *mprA*::Kan strains using primers (P1 and P2) shown in panel A. Download FIG S7, PDF file, 0.03 MB. Copyright © 2017 Kurthkoti et al. 2017 Kurthkoti et al. This content is distributed under the terms of the Creative Commons Attribution 4.0 International license.

### Proteomic analysis of human lung tissues.

The proteomic analyses of human lung tissues were described previously (19). Briefly, tubercular lung tissues were removed from participants who had undergone therapeutic lung resection surgery at the National Masan Hospital (NMH; South Korea). The patients had been infected with multidrug-resistant (MDR) TB and were treatment refractory to second-line drug therapy. The tissue collection has been described previously (73) and was approved by the NMH institutional review board, and an exemption for studying the archived tissues was granted by NIH, with written consent of the subjects; samples collected between 2002 and 2008 were deidentified when provided for dissection. The lung tissues were from a set of six participants and largely displayed three types of pathological structures, namely, solid cellular granulomas, caseous granulomas, and cavitary granulomas. To obtain histologically distinct compartments, necrotic (caseum) and cellular (cell) regions were dissected from each granuloma type with the Laser Pressure Catapulting (LPC) Palm instrument (Zeiss, Göttingen, Germany). Dissected tissues were lysed and subjected to in-gel digestion (Lys-C protease and trypsin) and peptide extraction. Liquid chromatography-tandem mass spectrometry (LC-MS-MS) analysis of resulting peptides was performed as single-shot runs, using a Q-Exactive mass spectrometer (Thermo Fisher Scientific) coupled online with a nanoflow ultra-high-pressure liquid chromatography (UHPLC) instrument (Easy nLC; Thermo Fisher Scientific). Protein quantification was performed as described previously (19, 74). Mass spectra were analyzed using the MaxQuant computational platform version 1.3.0.5 and Andromeda against the UniProt FASTA human database, and proteome quantification was performed in MaxQuant using the XIC-based inbuilt label-free quantification (LFQ) algorithm as previously described (19).

The abundance of proteins was based on the relative quantification of a protein in a given dissected histological region compared to all other samples subjected to proteomics. The normalization utilized in the label-free quantification exploits the fact that there are dominant populations of proteomes that do not change between experimental conditions or samples, so that their average behavior can be used as a relative standard. Thus, the abundance is determined relative to a standard that is derived from common peptides/proteins that change minimally between experimental conditions.

We analyzed proteins associated with Fe metabolism from these data that are publicly available and have been deposited into the PRIDE partner repository with the data set identifier PXD003646, which can be accessed from the Proteome Xchange Consortium (http://proteomecentral.proteomexchange.org). The heat map plot was constructed from Z score- and log_2_-transformed LFQ protein intensities.

### Analyses of host Fe metabolism gene expression in human granulomas.

Expression of Fe homeostasis host genes in the cellular region adjacent to necrotic-cavitary granulomas was analyzed in comparison to uninvolved tissue isolated from the lungs of human TB patients from publicly available data (GEO accession number GSE20050). Acquisition of lung tissue, processing of the samples, and DNA microarray analysis were reported previously (27).

### Iron starvation procedure.

To minimize Fe contamination from glass, plastic containers were exclusively used. An exponentially growing *M. tuberculosis* strain revived from a frozen stock on a 7H10 agar plate was inoculated into 5 ml of MM at an initial optical density at 540 nm (OD_540_) of 0.1 in 15-ml polypropylene tubes and incubated at 37°C, with constant agitation, in a cell culture rotator to early stationary phase (OD_540_ of ~1). MM cultures were diluted to an OD_540_ of 0.1 in the same medium and allowed to grow to early stationary phase to deplete stored Fe and then diluted to an OD_540_ of 0.1 in MM containing 50 μg ml^−1^ of freshly prepared deferoxamine mesylate (Sigma). This culture replicated slowly and reached a maximum OD_540_ of 0.35 to 0.40. At this point, the culture was divided in two and diluted to an OD_540_ of 0.1 in the same medium or in MM containing 50 µM FeCl_3_ (Fe-replete culture). The MM+DFO culture ceased to grow, and therefore, it was maintained for phenotypic studies while the Fe-replete culture was collected 24 h afterward and used as a reference for microarray and metabolite analyses. The growth-arrested MM+DFO cultures are referred to here as Fe-starved growth-arrested *M. tuberculosis* (ISGAM).

### Determination of cell viability.

At various time points, 10-fold serial dilutions of ISGAM cultures were made and plated onto 7H10 agar to enumerate CFU. Live/dead staining with fluorescein diacetate (FDA) and propidium iodide was used to distinguish living cells from the dead-cell population.

For FDA and PI staining, 1 ml of culture was centrifuged, and the pellet was resuspended in 1 ml of phosphate-buffered saline (PBS)–0.02% Tween 80 and stained with FDA (final concentration, 10 µg ml^−1^) and PI (final concentration, 4 μg ml^−1^) for 20 min, at room temperature, in the dark. Samples were centrifuged and washed once with PBS-0.02% Tween 80, fixed with 8% paraformaldehyde, and concentrated to 0.1 ml by centrifugation. Five microliters of the sample was added to a 1% agarose pad, placed on a poly-d-lysine-coated Fluorodish (Time Precision Instruments), and visualized on an Axiovert 200 M inverted fluorescence microscope (Zeiss), equipped with a CoolSNAP HQ camera (Photometrics, Pleasanton, CA). Images were acquired with OPENLAB acquisition software (Improvision, Sheffield, United Kingdom).

### Recovery from Fe starvation in liquid medium.

At the desired time point, DFO was removed from Fe-starving cultures by centrifugation. The bacterial pellet was resuspended in MM supplemented with 80 μM FeCl_3_ or MM-DFO as a control and incubated at 37°C with agitation. Growth was monitored by measuring the increase in optical density.

For most probable number (MPN) determinations, 10-fold serial dilutions from three independent cultures were made up to a 10^−7^ dilution and 100 µl of each dilution was added to 1.9 ml 7H9 in a 5-ml polystyrene round-bottom tube. For each dilution, there were five replicate tubes. The tubes were incubated at 37°C for 2 months. Tubes with visible turbidity were counted as positive, and MPN was calculated using standard statistical methods (31).

### Antibiotic sensitivity assays.

Sequential dilutions of ISGAM and Fe-sufficient *M. tuberculosis* cultures were treated for 3 days with increasing concentrations of several antibiotics. After treatment, the cultures were serially diluted in 7H9 and 5 μl of each dilution was spotted on 7H10 plates. Cells not treated with antibiotics were also spotted in the same plates as a control. We report MBC_90_, which is the concentration of drug that resulted in 90% reduction in the CFU of the treated culture compared to the untreated control.

### RNA extraction and bacterial transcriptomic analyses.

ISGAM cultures were collected at 1, 7, and 14 days, Fe-replete cell cultures were collected at day 1 and centrifuged at 4,500 rpm for 5 min, and the cell pellets were resuspended in 1 ml TRI reagent and immediately transferred to a tube containing 0.5 ml of 200-µm zirconia beads (Sigma-Aldrich) and disrupted by two 1-min pulses in a BeadBeater. RNA was purified as previously described (75) using RNeasy columns according to the manufacturer’s instructions (Qiagen). The quality and quantity of purified RNA were estimated using a NanoDrop spectrophotometer (NanoDrop, Wilmington, DE). Total RNA was isolated from triplicate cultures and three independent experiments.

Microarray analysis was performed as previously described (76). The *M. tuberculosis* DNA microarray consisted of 4,295 70-mer oligonucleotides representing the 3,924 predicted open reading frames (ORFs) of the H37Rv strain (http://www.sanger.ac.uk). The arrays were prepared by spotting oligonucleotides (tuberculosis genome set version 1.0; Operon Biotechnologies) onto poly-l-lysine-coated glass microscope slides, using a GeneMachines Omnigrid 100 Arrayer (Genomic Solutions) and SMP3 pins (Telechem) as described above. Briefly, cDNA was synthesized using random primers and labeled with Cy3- or Cy5-dUTP (PerkinElmer) as described before (48) and hybridized to the arrays overnight. After washing, the arrays were scanned with a GenePix 4000B scanner (Molecular Devices). The images were processed using GenePix 5.1. Data were filtered by removing all spots that were not above the background noise. Spots were considered to be not sufficiently above background noise for further analysis if the sum of the median intensities of the two channels was less than twice the highest mean background of the chip. The chips were normalized by the print-tip LOWESS method (77). The ratio of the mean median intensity of Cy5 to the mean median intensity of Cy3 was determined for each spot, and the fold change values were calculated.

### RNA sequencing.

RNA from biological triplicates of ISGAM cultures at 1 and 7 days and from Fe-replete *M. tuberculosis* cultures was isolated using the Directzol RNA extraction kit (Zymo Research) according to the manufacturer’s instructions. DNA libraries were prepared from total RNA using the RNAtag-Seq protocol (78). Briefly, RNA samples were fragmented, depleted of genomic DNA, and dephosphorylated prior to their ligation to barcoded adaptors. Barcoded RNAs were pooled, depleted of rRNA using RiboZero (Epicentre), and converted to Illumina cDNA libraries in 3 steps: (i) reverse transcription of the RNA primed from the ligated adaptor, (ii) degradation of the RNA and ligation of a second adaptor to the single-stranded cDNA, and (iii) PCR amplification of the cDNA with primers targeting the ligated adaptors and carrying the full sequence of the Illumina sequencing adaptors. Libraries were sequenced on HiSeq 2000 to yield 25-base paired-end (PE) reads.

### RNA-sequencing data analysis.

Sequencing reads were aligned to the H37Rv genome (RefSeq NC_000962). The overall fragment coverage of genomic regions corresponding to features such as open reading frames and rRNAs based on RefSeq annotations was determined by using bioinformatic pipelines developed in-house as previously described (79). Differential expression analysis was conducted with DESeq (80).

### Metabolite extraction and analysis.

Metabolite extraction was performed as described previously (81). Briefly, cultures containing ~5 × 10^8^ bacteria were quenched in 10 ml of 100% methanol at 4°C, spun down, and resuspended in acetonitrile-methanol-water (2:2:1). Cells were lysed mechanically by using a bead beater (MP Biomedicals) and spun down, and metabolites were removed and filtered. Metabolite content was analyzed by UPLC-coupled mass spectrometry (Waters, Manchester, United Kingdom). Column eluents were delivered via electrospray ionization. UPLC was performed in a hydrophilic interaction liquid chromatography-mode gradient elution using an Acquity 1.7-μm amide column (2.1 by 150 mm) based on a method previously described (82). The flow rate was 0.5 ml/min with mobile phase A (100% acetonitrile) and mobile phase B (100% water), both containing 0.1% formic acid. In both positive and negative modes, the gradient began with 1% B until 1 min, was ramped to 35% B by 14 min and then 60% B by 17 min, was held at 60% B for 1 min, was then ramped to 1% B by 19 min, and was held at 1% B to the end of the run at 20 min. The mass spectrometer was operated in V mode for high sensitivity using a capillary voltage of 2 kV and a cone voltage of 17 V. The desolvation gas flow rate was 500 liters/h, and the source and desolvation gas temperature were 120°C and 325°C, respectively. MS spectra were acquired in centroid mode from *m/z* 50 to 1,200 with a scan time of 0.5 s. Leucine enkephalin (2 ng/μl) was used as lock mass (*m/z* 556.2771 and 554.2615 in positive and negative experiments, respectively). A TargetLynx (Waters) database was compiled by running standards of metabolites of interest to determine retention times and *m/z*. All samples were analyzed using TargetLynx (Waters) with manual curation of peak areas where necessary. Peak areas were normalized to number of cells and used to calculate fold changes of metabolites relative to Fe-replete cultures. When peaks were not detected, the peak area was set to 1 to calculate fold changes. Metabolite abundances, retention times, and *m/z* values can be found in Data Set S2.

10.1128/mBio.01092-17.10 DATA SET S2  Metabolite abundances in extracts of Fe-starving *M. tuberculosis*. Download DATA SET S2, XLSX file, 0.02 MB. Copyright © 2017 Kurthkoti et al. 2017 Kurthkoti et al. This content is distributed under the terms of the Creative Commons Attribution 4.0 International license.

### Statistical analyses.

All experiments were performed in triplicate and repeated on at least two different occasions. Data are expressed as means ± standard deviations (SD). Differences between frequencies were assessed by Student’s *t* test (bilateral and unpaired) using a *P* value of <0.05 as statistically significant. In the microarray analyses, a one-class SAM analysis (83) was performed with the MEV software (84) to find genes with changes that occurred consistently in all replicates. A median false discovery rate (FDR) of zero, delta values ranging from 3.14 to 3.63, and a mean change of at least 2-fold were considered our cutoff for significance. Metabolite analysis was performed using MarkerLynx (Waters) with extended statistic function to identify statistically significant differences of metabolite abundances.

### Data availability.

The microarray data discussed in this publication have been deposited in NCBI’s Gene Expression Omnibus and are accessible through GEO series accession number GSE84554.
