# Supplementary material for: The Capacity of Mycobacterium tuberculosis To Survive Iron Starvation Might Enable It To Persist in Iron-Deprived Microenvironments of Human Granulomas
Source: mBio. 2017 Aug 15;8(4):e01092-17. doi: 10.1128/mBio.01092-17 (PMC5559634; doi:10.1128/mBio.01092-17)
Supplement: FIG S1 [file mbo004173421sf1.pdf]

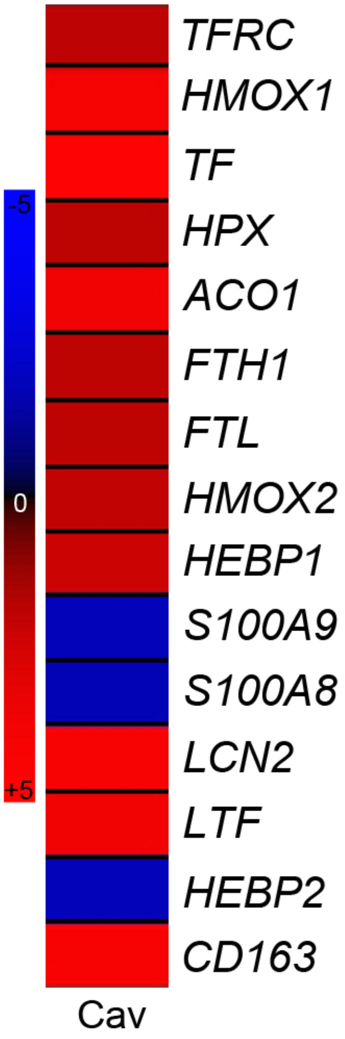

Supplementary Figure 1. Host Fe-metabolism gene expression in TB cavitary granulomas. Heat map representing abundance of transcripts for genes encoding the indicated Fe-metabolism proteins in the cellular region adjacent to necrotic-cavitary (Cav) granulomas compared to uninvolved regions isolated from the lungs of human TB patients. TFRC: Transferrin; HMOX1 and 2: Heme oxygenase; TF: transferrin; HPX: hemopexin; ACO1 aconitase; FTH1: Ferritin heavy chain; FTHL: Ferritin light chain; HEPB1 and 2: Heme binding protein; S100A9/8: Calprotectin; LCN2: Lipocalin; LTF: Lactoferrin; CD163: Haptoglobin-hemoglobin receptor. Acquisition of lung tissue and processing of the tissue for microarray analysis is described in Materials and Methods.
