# Supplementary material for: The Capacity of Mycobacterium tuberculosis To Survive Iron Starvation Might Enable It To Persist in Iron-Deprived Microenvironments of Human Granulomas
Source: mBio. 2017 Aug 15;8(4):e01092-17. doi: 10.1128/mBio.01092-17 (PMC5559634; doi:10.1128/mBio.01092-17)
Supplement: FIG S2 [file mbo004173421sf2.pdf]

A. B.

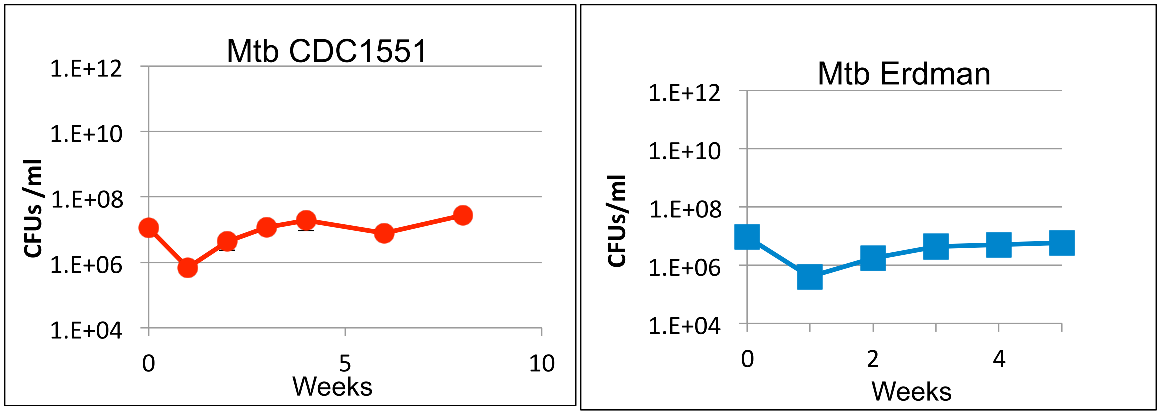

Supplementary Figure 2. **A.** *Mtb* CDC1515 and **B**. *Mtb* Erdman persistence under Fe-starvation. Shown is the number of CFUs/ml recovered from MM+DFO cultures. Data are expressed as the mean ± standard deviations from three biological replicates.
