# Supplementary material for: The Capacity of Mycobacterium tuberculosis To Survive Iron Starvation Might Enable It To Persist in Iron-Deprived Microenvironments of Human Granulomas
Source: mBio. 2017 Aug 15;8(4):e01092-17. doi: 10.1128/mBio.01092-17 (PMC5559634; doi:10.1128/mBio.01092-17)
Supplement: FIG S3 [file mbo004173421sf3.pdf]

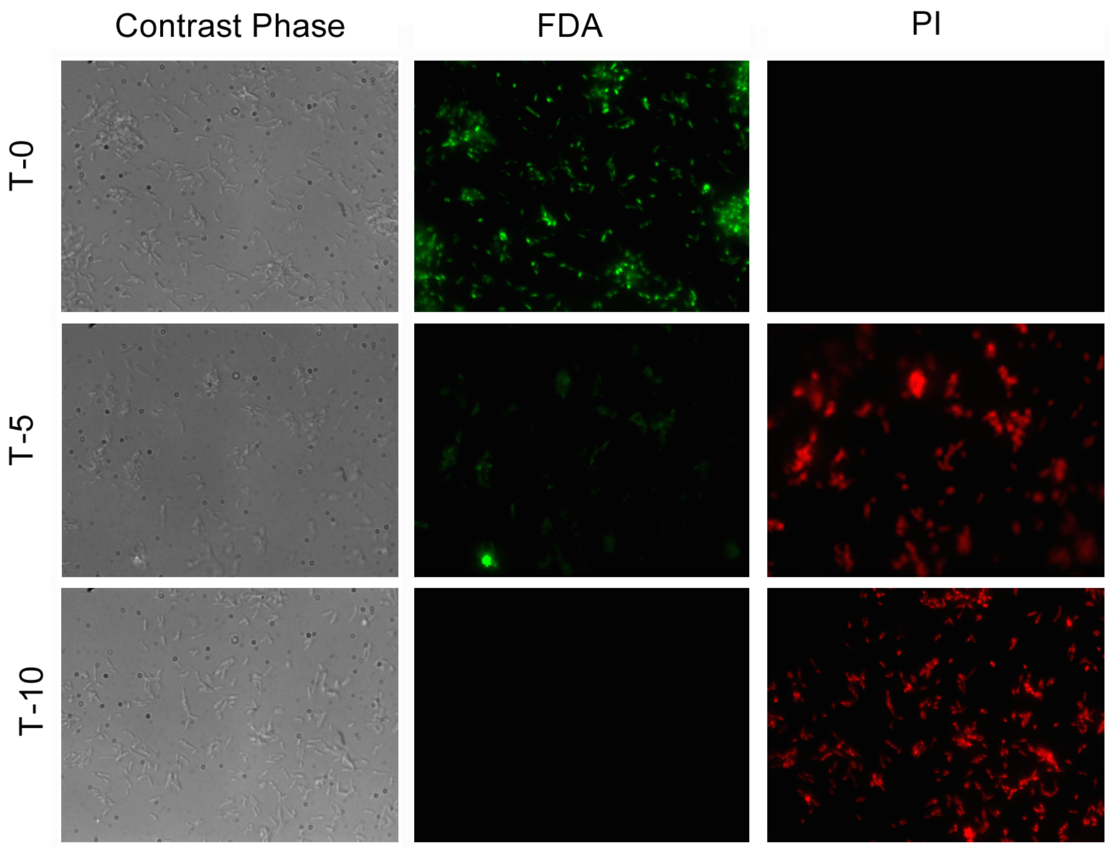

Supplementary Figure 3. Validation of FDA/PI viability staining.
Mycobacterial cells were subjected to 80°C for 0, 5 or 10 minutes (T-0,5,10) and stained with FDA or PI. While unstressed viable cells stain with FDA they do not stain with PI. Heat killed cells are stained by PI but not by FDA.
