# Supplementary material for: The Capacity of Mycobacterium tuberculosis To Survive Iron Starvation Might Enable It To Persist in Iron-Deprived Microenvironments of Human Granulomas
Source: mBio. 2017 Aug 15;8(4):e01092-17. doi: 10.1128/mBio.01092-17 (PMC5559634; doi:10.1128/mBio.01092-17)
Supplement: FIG S4 [file mbo004173421sf4.pdf]

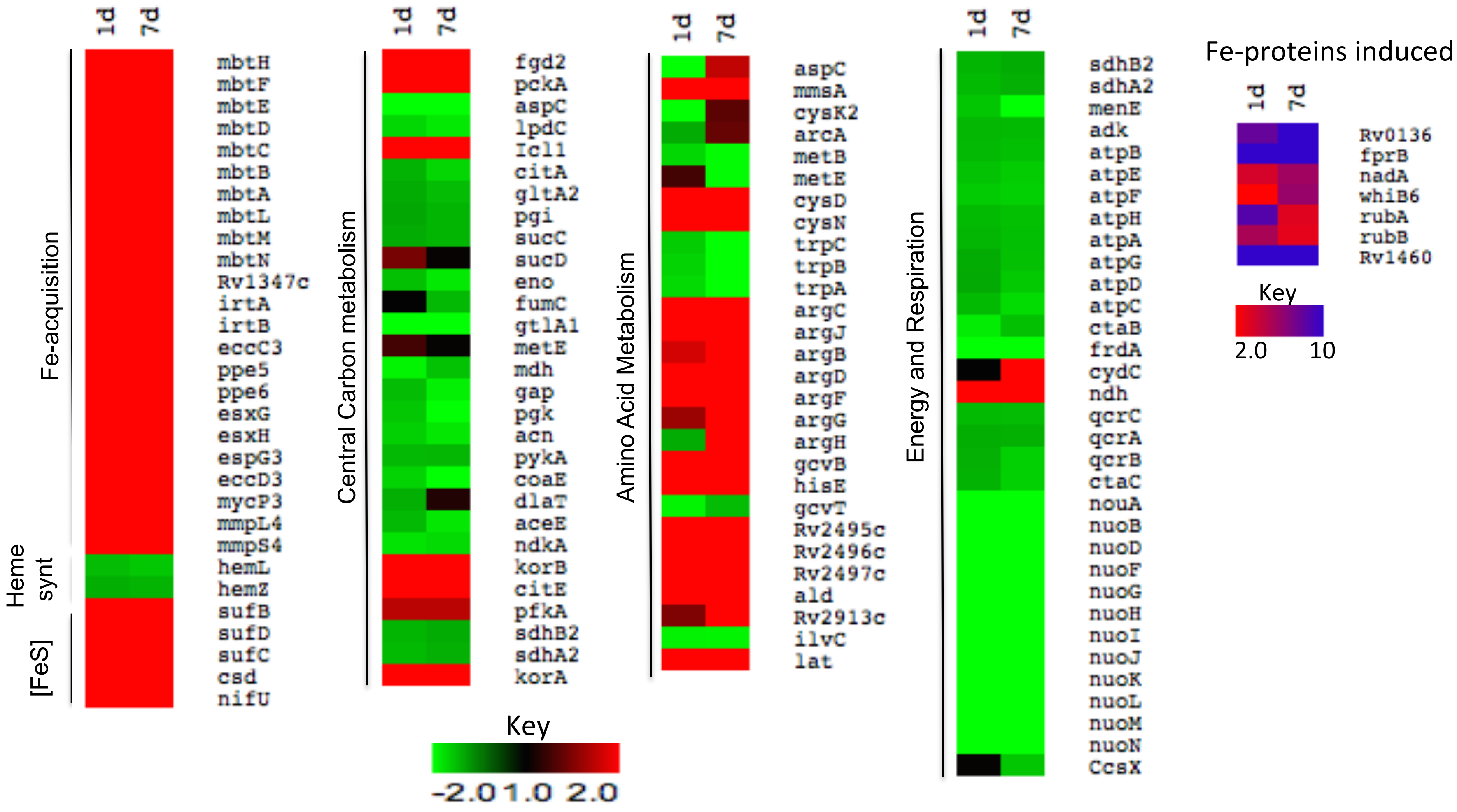

Supplementary Figure 4. Heat map based on RNA-seq of selected differentially expressed genes detected in microarrays. Triplicate cultures were harvested at day 1 and 7 and RNA was extracted and process for RNA-seq as described in materials and methods to validate the microarrays. P value adjusted for multiple testing using Benjamin-Hochberg procedure (P_{adj} is shown in Supplementary Data set 4.
