# Supplementary material for: The Capacity of Mycobacterium tuberculosis To Survive Iron Starvation Might Enable It To Persist in Iron-Deprived Microenvironments of Human Granulomas
Source: mBio. 2017 Aug 15;8(4):e01092-17. doi: 10.1128/mBio.01092-17 (PMC5559634; doi:10.1128/mBio.01092-17)
Supplement: TABLE S1 [file mbo004173421st1.pdf]

Supplementary Table 1. Functional categories of genes upregulated during the Fe-starvation response (ISR).

Functional Categories	ISR No. genes			%ISR			%genome
	1d	7d	14d	1d	7d	14d	
intermediary metabolism and respiration	55	83	51	26.9	22.1	20.5	22.4
conserved hypotheticals/unknown	50	118	75	24.5	31.7	30.2	31.9
lipid metabolism	36	23	23	17.6	6.1	9.2	5.9
cell wall and cell processes	27	44	21	13.2	11.7	8.4	18.8
information pathways	4	12	8	1.9	3.2	3.2	5.8
regulatory proteins	17	40	28	8.3	10.6	11.2	4.8
PE/PPE	8	17	7	3.9	4.5	2.8	4.2
insertion sequences and phages 5	1	14	11	0.5	3.7	4.4	3.7
virulence, detoxification, adaptation 0	6	24	24	2.9	6.4	9.6	2.6
