# Supplementary material for: The Capacity of Mycobacterium tuberculosis To Survive Iron Starvation Might Enable It To Persist in Iron-Deprived Microenvironments of Human Granulomas
Source: mBio. 2017 Aug 15;8(4):e01092-17. doi: 10.1128/mBio.01092-17 (PMC5559634; doi:10.1128/mBio.01092-17)
Supplement: FIG S5 [file mbo004173421sf5.pdf]

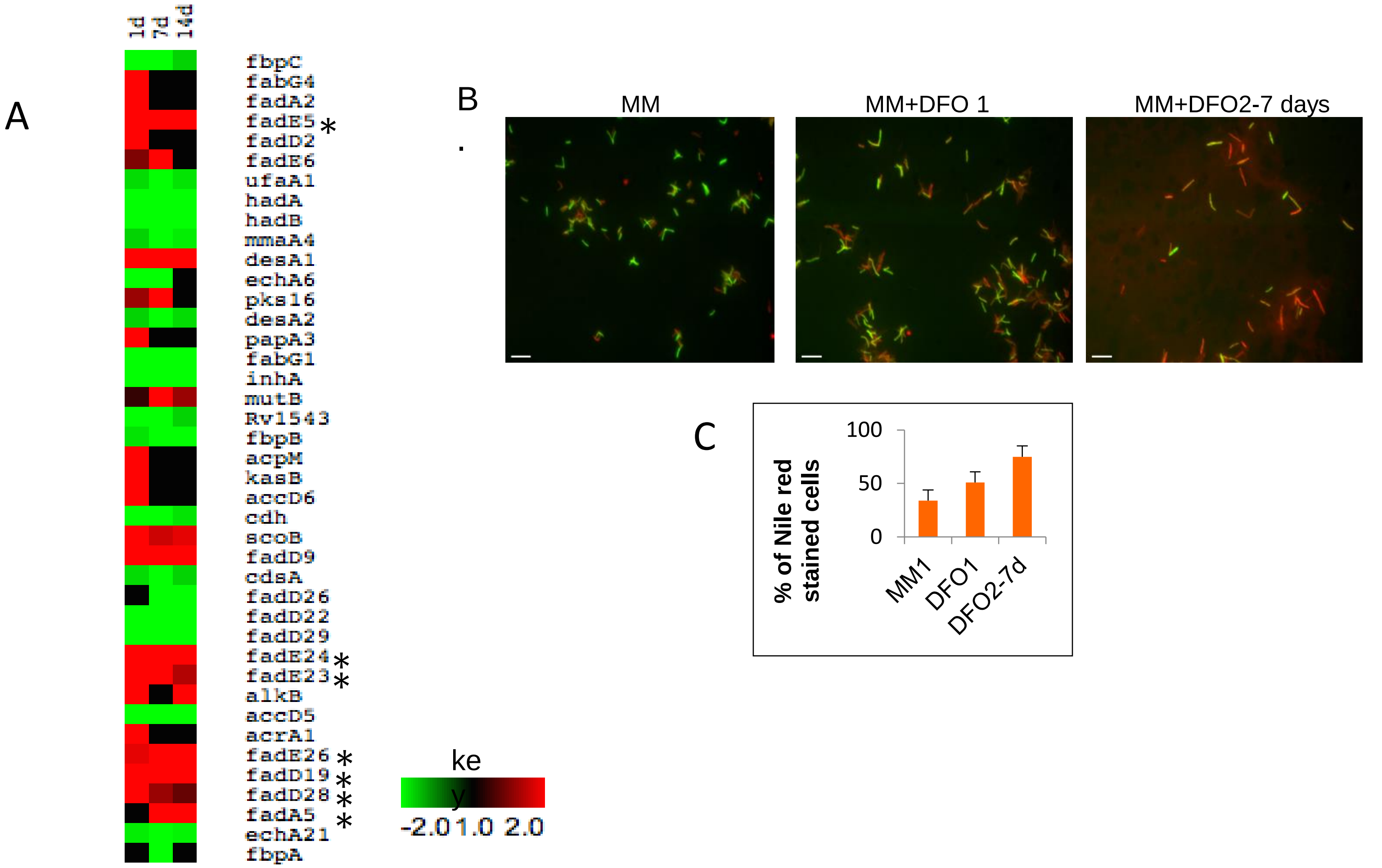

Supplementary Figure 5. A. Heat map transcriptional profile of lipid metabolism genes in Fe-starved *Mtb*. Triplicate cultures from three independent experiments were harvested at days 1, 7 and 14, and RNA was extracted. Genes involved in cholesterol catabolism are marked with *. P values for heat maps can be found in the GEO database (accession no. GSE84554). **B**. Microscopic images representative of Auramine and Nile Red staining of Fe-starving *Mtb* at indicated time points MM: first passage in MM; MM+DFO1: first passage in MM+DFO; MM+DFO2: Second passage in MM+DFO.**C**. Percentage of Nile red stained cells in at least 500 cells counted.Data are expressed as the mean ± standard deviations from three biological replicates
