# Supplementary material for: The Capacity of Mycobacterium tuberculosis To Survive Iron Starvation Might Enable It To Persist in Iron-Deprived Microenvironments of Human Granulomas
Source: mBio. 2017 Aug 15;8(4):e01092-17. doi: 10.1128/mBio.01092-17 (PMC5559634; doi:10.1128/mBio.01092-17)
Supplement: FIG S6 [file mbo004173421sf6.pdf]

A.   B.

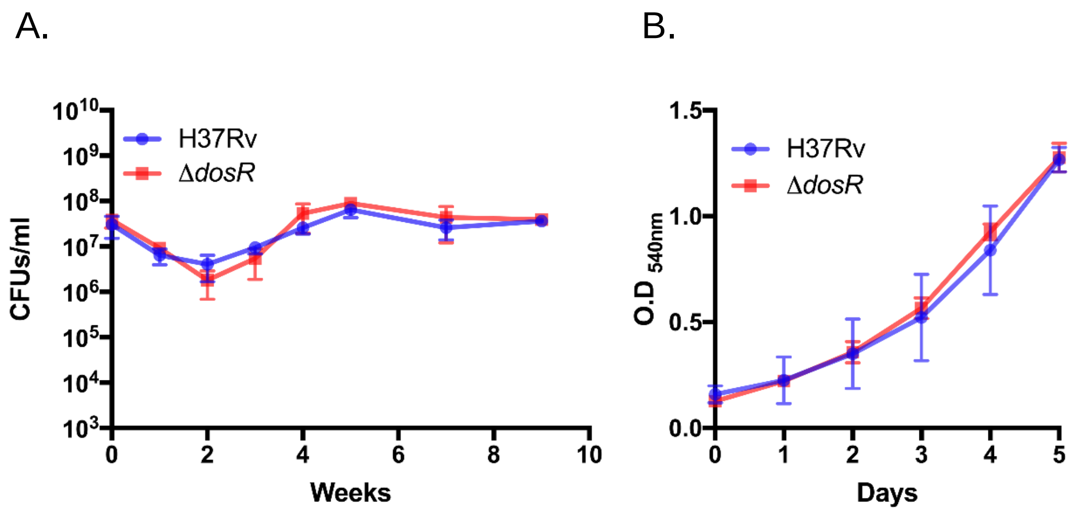

Supplementary Figure 6. Survival and recovery of a *dosR* mutant subjected to Fe-starvation. A. Shows the CFUs recovered from MM+DFO cultures of wild type and Δ*dosR* mutant at indicated time points into Fe-starvation. **B**. Shows growth measured as the increase in O.D at 540nm of wild type and Δ*dosR* when provided with $FeCl_3$. Error bars represent mean ± standard deviations from three biological replicates.
