# Supplementary material for: The Capacity of Mycobacterium tuberculosis To Survive Iron Starvation Might Enable It To Persist in Iron-Deprived Microenvironments of Human Granulomas
Source: mBio. 2017 Aug 15;8(4):e01092-17. doi: 10.1128/mBio.01092-17 (PMC5559634; doi:10.1128/mBio.01092-17)
Supplement: FIG S7 [file mbo004173421sf7.pdf]

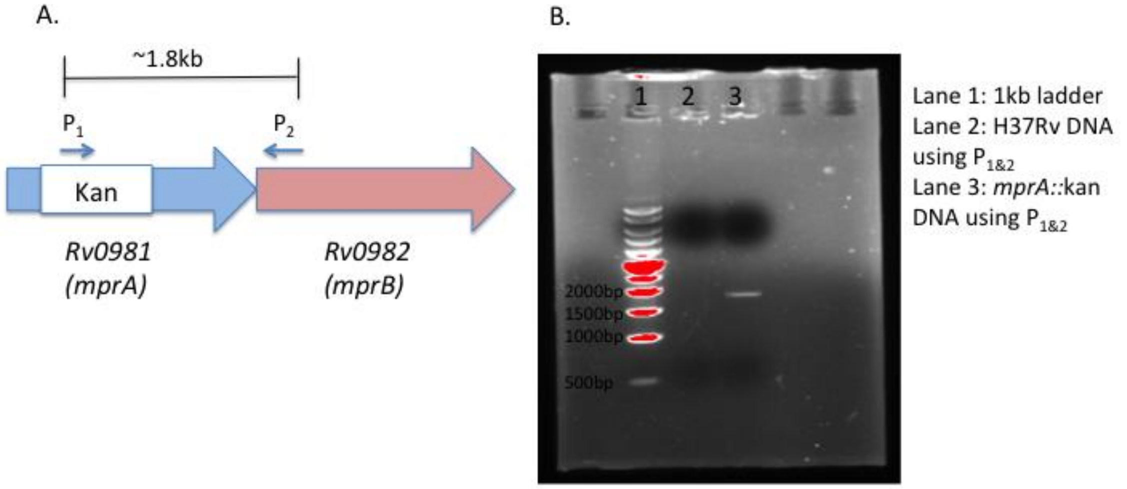

Supplementary Figure 7. Confirmation of *mprA* knock out mutant strain. A. Shows the schematic of the insertion of kanamycin cassette within *mprA.* **B**. Shows PCR amplification in the wild type and *mprA*::kan using primers (P_1 & P_2) shown in **A**.
